# Supplementary material for: Are carbon emissions trading and green financial instruments synergistic? -Comprehensive quantitative research based on content analysis
Source: PLoS One. 2024 Mar 7;19(3):e0298601. doi: 10.1371/journal.pone.0298601 (PMC10919601; doi:10.1371/journal.pone.0298601)
Supplement: S1 Data — (ZIP) [file pone.0298601.s001.zip › Data/地级市Z_F工作报告.zip!/╝¬┴╓/╝¬┴╓╩╨/└┤╫╘╣1⁄2╓┌║┼í╛┬φ┐╦╩2╛▌═°í┐_2013.docx]

各位代表：

　　现在，我代表市政府向大会报告工作，请予审议，并请政协委员提出意见。

　　一、过去五年及2012年工作回顾

　　五年来，我们在省委、省政府和市委的正确领导下，在市人大、市政协的大力支持下，团结带领全市人民，牢牢把握科学发展、转变发展方式的主题主线，积极应对金融危机冲击、特大洪涝灾害等诸多严峻困难和挑战，统筹“三化”建设、落实“三动”战略，全力实施经济总量翻番等重大部署，圆满完成了本届政府确定的各项目标任务，振兴老工业基地取得了重大成就，吉林大地发生了巨大的历史性变化。

　　——过去五年，是经济实力显著增强的五年。2012年，实现地区生产总值比2007年增长1.7倍（预计数，下同），年均增长21.7%，占全省份额提升到22%；规模工业增加值增长1.9倍，年均增长24.1%；全口径财政收入增长2倍，年均增长24.4%；地方级财政收入增长2.2倍，年均增长26.1%；固定资产投资累计比前5年增长2倍，年均增长24.6％；三次产业结构由13.1∶49.6∶37.3调整到9.2∶50.7∶40.1。

　　——过去五年，是改革开放成果丰硕的五年。扎实推进重点领域和关键环节改革，政府机构改革和行政审批制度改革不断深化，企业改革稳步实施，农村综合改革成效明显，集体林权制度改革基本完成，医药卫生体制改革取得阶段性成果。开放型经济达到新水平，累计招商引资到位资金1940亿元，实际利用外资24.6亿美元，外贸进出口总额55.5亿美元。长吉一体化强力推动，开发区产业集聚和开放带动作用明显增强，友好城市和友好经贸合作城市发展到68个。

　　——过去五年，是城乡面貌明显改善的五年。“北工、中商、南居”的城市空间结构形态初步形成，中心城市建成区面积扩大到167平方公里。城市功能日益完善，环境保护和生态建设成效显著，被评为中国十大最美丽城市、十大休闲城市。城乡发展协调性增强，县域生产总值、地方级财政收入分别年均增长21.2%和28.8%；桦甸、磐石跨入全省县域发展综合实力前十名，一批节点镇、中心镇特色发展优势突出，新农村建设走在全省前列。

　　——过去五年，是民生事业长足发展的五年。坚持每年落实一批民生事项和富民惠民工程，累计支出500亿元，一大批涉及广大群众切身利益的现实问题得到妥善解决。城乡就业稳步扩大，劳动关系更加和谐；社会保障体系日趋完善，覆盖范围不断拓展；城乡低保标准持续提高，社会救助体系进一步健全；衣食住行条件明显改善，家庭财产稳定增加，城镇居民人均可支配收入、农民人均纯收入分别年均增长12.7%和15.5%。

　　——过去五年，是社会事业全面进步的五年。科技创新能力明显增强，科技研发和科技成果转化成效显著，完成市级以上科技计划项目950项，国家碳纤维产业基地建设取得重大进展。教育优质、均衡发展步伐加大，基础教育、职业教育保持全省领先，高等教育健康发展。文化事业和文化产业蓬勃发展，公共卫生保障能力和医疗服务水平有力提升，人口和计生、广播电视、民族团结进步创建活动获得国家表彰，连续七次被评为全国双拥模范城。平安吉林建设成果持续巩固和加强。

　　各位代表，五年来，吉林市走过了极不平凡的发展历程。特别是本届政府任期的最后一年，面对外部环境总体趋紧、经济下行压力增大、一些重点行业和骨干企业受到较大影响的复杂形势，我们把稳增长作为核心任务，全力实施“十项攻坚战”，经济社会保持了平稳较快的发展态势。预计可完成地区生产总值2690亿元，同比增长21.8%；规模工业增加值970亿元，增长20.9%；招商引资到位资金790亿元，增长33.9%；固定资产投资2000亿元，增长33%；社会消费品零售总额1010亿元，增长25.6%；全口径财政收入300亿元，增长22.5%；地方级财政收入130亿元，增长27.6%；城镇居民人均可支配收入22688元，农民人均纯收入9224元，均增长16%。

　　一是大力开展项目建设年活动，招商引资和项目建设成效明显

　　围绕扩总量、调结构，强化项目谋划，组织开展集中开工、“大巡检”等系列活动，项目建设数量、质量大幅提升，实施3000万元以上重点项目1100项，同比增加224项。其中，亿元以上580项、5亿元以上125项、10亿元以上50项。30万吨环氧丙烷、年产5000辆商品运输车等550个重点工业项目开工建设，吉化40万吨ABS（一期）、一汽吉林扩能改造G3、中钢500吨碳纤维等一批重点项目建成投产；现代服务业项目投资比重提升，社会民生、基础设施、交通能源等领域项目建设成效明显，项目向十大功能区、各类开发区及园区集聚的趋势日益鲜明。积极争取国家稳增长政策，丰满水电站重建工程开工，国电吉林热电厂扩建、小城钼矿开发等项目获得国家批复。

　　扩大招商主体，强化责任落实，高密度组织开展多轮境内外招商活动，中新吉林食品区合作项目正式签约，新加坡吉宝讯通、美国微软、韩国乐天等跨国公司落户我市，万丰工业园、增益物流园等一批重大项目落地建设，全年新开工投资3000万元以上招商项目350项，其中亿元以上项目160项；实际利用外资7.1亿美元，增长15%。多元拓宽融资渠道，华微电子等公司债券成功发行，城投、铁投、土地收储中心等平台融资规模扩大，兴业、民生、中信银行分行正式运营，新增19户小额贷款公司和2户担保公司，10家基金及管理公司完成设立，采取BT等多种模式实现融资340亿元。抢抓国家实施新一轮老工业基地调整改造机遇，充分利用国家节能减排财政政策综合示范城市、电子商务示范城市、城市矿产示范基地等“帽子”优势，争取国家和省各类财政资金128.5亿元。

　　二是突出空间布局优化，功能区建设和县域发展步伐加大

　　举全市之力加快十大功能区开发建设，相关规划编制完成，各功能区基本定位和发展目标更加明晰；着力推动项目进区，实施基础设施项目80项、产业项目300项。高新南区、经济开发区、化工循环经济示范园区的整车及零部件生产、碳纤维产业化、丙烯产业链等一批重大项目加快建设，主导产业集群发展优势凸显；高新北区强化对外合作，与中科院、清华大学合作协议正式签订，多渠道融资加快基础设施建设；哈达湾服务业集中区冀东水泥等企业搬迁有序推进；金珠工业区晨鸣纸业新厂主体框架完工，钢铁产业链进一步延伸；南部新城以欧亚城市综合体为代表的现代服务业项目大力推动；松花湖旅游区优化资源整合，万科国际旅游度假区项目开工建设；北大壶开发区雪上项目综合训练中心二期主体竣工，冰雪运动优势进一步彰显；中新两国合作的吉林食品区建设取得突破性进展，无规定疫病区通过农业部验收，国际物流园等一批重点项目启动实施。

　　强力推进县域发展，县域生产总值、地方级财政收入分别增长21.5%和29.6%。县域开发区基础设施不断完善，无机粉体环保纸、修正健康产业园等93项3000万元以上工业项目入区建设，蛟河、舒兰、明城工业集中区晋升为省级开发区。县城综合服务功能快速提升，建成区面积拓展到86平方公里；岔路河、夹皮沟、烟筒山、天岗、吉舒等长吉节点镇、中心镇产业支撑、基础设施和外在形象发生新的变化。

　　三是推进产业结构调整，经济增长协调性有效提高

　　工业经济保持平稳发展。充分发挥亿元专项资金的杠杆撬动作用，强化产需衔接和银企对接，在严峻多变的市场环境下完成规模工业产值3100亿元，增长12.1%；实现利润55亿元。民营经济成为推动发展的重要支撑力量，主营业务收入、实缴税金分别增长29%和17%，就业贡献率达到80%以上；中小企业成长迅速，新增规模工业企业105户，纳税超千万元民营企业增加到60户。众鑫异戊烯醇、长白山康艾注射液等46项3000万元以上战略性新兴产业项目建成投产。推进147项重点工业项目达产达效，达产达效率达到81%，新增产值170亿元。超额完成省下达的节能减排任务。

　　服务业发展水平明显提升。设立服务业引导资金，努力创造支持服务业发展的社会环境，第三产业增加值增长24%。商贸经济加快发展，红星美凯龙家俬城、凯西隆商贸中心等42个重点项目开工建设，紫光苑酒店等7个项目投入运营；限额以上商贸企业发展到1033户，新增229户；组织开展多种营销活动繁荣市场，城乡商贸流通体系进一步完善。文化旅游产业融合发展，建成73项文化旅游产业项目，雾凇冰雪节、松花江河灯文化节、蛟河红叶节等节庆活动影响力不断扩大，旅游总收入增长26%。生产性服务业发展迅速，博宇医药、荣德钢铁等物流项目建设步伐加大；中油、软银等大型数据处理中心项目启动，微软IT培训学院落户，大仝数码等服务外包企业成长壮大；科技服务、市场中介等实现较快发展。

　　现代农业稳步发展。粮食产量突破110亿斤，再创历史新高。畜牧业、特产业规模扩大，产值分别增长8%和21.9%。生态林业建设取得新成果。建设中粮稻米深加工基地等一批重点农业产业化项目，龙升长白山特产加工等10个项目建成投产，市级以上龙头企业发展到200户。农业综合生产能力增强，土地整治成效明显，实施各类农田水利建设项目504项，农业综合机械化水平达到68%，三级农业信息服务平台全部建成，新发展农民专业合作社300个。集体经济收入超百万元村达22个，20万元村达121个。

　　四是加强城市建设与管理，城市综合服务功能快速增强

　　完成城建投资251亿元，房地产业和建筑业增加值分别增长22.3%和42.3%。新建续建秀水大桥、雾凇西路等231项重点城建项目，江城大桥、松花江拦江壅水坝等108项投入使用。长吉北线一级公路改建项目开工，绕城高速西南环段通车，建成各级各类公路435.5公里。房地产开发1070万平方米，竣工700万平方米。维修改造22项道桥工程，新建供水管线3条，铺设天然气管线35公里。大力开展城市管理提升年活动，广泛发动市民参与城市管理，小区环境、牌匾广告等专项整治行动收到实效，重点街路建筑物“穿衣戴帽”、移植乔木等一批美化绿化工程高标准实施，一江两岸夜色景观更趋鲜明，城市整体形象和市容环境得到明显改善。

　　五是加大改善民生力度，社会民生事业成果显著

　　十大方面150项民生事项全部完成，总投入达到145亿元。城镇新增就业9.8万人，农村劳务输出65万人次。棚户区和危旧房改造建设回迁住房98万平方米，新建廉租房、公租房30.6万平方米，3.4万户居民享受到租赁补贴。暖房子工程建造热网306公里，完成居住建筑节能改造1246万平方米，17.7万户家庭直接受益。510种基本药物零加价在全市基层医疗卫生机构实施，城镇居民医疗保险、新农合报销最高支付限额分别提高到6万元和8万元。城乡困难群众医疗救助自理费用降到22%以下。城市低保标准提高到每人每月330元、农村低保标准达到每人每年2000元。为全市低保家庭在校中小学生提供了免费午餐。在全省率先开展城市放心早餐工程试点，建立平价副食品商店35家。取消了城区露天占道早夜市收费。又有16万农村人口安全饮水问题得到解决。建成2400栋温室大棚。新建社区居家养老服务站62个。更新公交车辆150台。扎实开展“一下四送”活动，18项城区低保困难家庭再保障政策深入实施，一批涉及弱势群体和困难群众就业、上学、就医、住房等问题得到较好解决。

　　科技创新取得重要成果，实施市级以上重大科技成果转化和科技攻关项目220项，与中科院等高校院所产学研合作深入开展。积极推进城乡教育优质均衡发展，调整撤并中小学校67所，完成80所校舍建设改造；学前教育普及进程加快，素质教育水平提升，职业教育取得新的发展成绩。基层医疗卫生机构改革成果不断巩固，公立医院改革试点稳步实施；区域医疗中心和社区卫生服务中心功能逐步完善，农村三级医疗网络建设进一步加强。文化体育事业亮点纷呈，市歌舞团持续占领国内高端演出市场，流动河灯施放再创吉尼斯世界纪录，《水姑娘》、《牛子厚与喜连成》等剧目获国家级最高奖，被评为中国书法城，成功承办第十二届全国冬运会雪上赛事并取得历史最好成绩，广大群众的精神文化生活更趋丰富多彩。

　　六是加快转变政府职能，民主法制建设进一步加强

　　主动接受市委领导，自觉接受人大、政协监督，认真吸纳公众意见建议，健全重大事项民主决策机制，政府决策的科学性有效增强。六五普法规划深入实施，制定地方性法规（草案）和政府规章5部，行政行为进一步规范，法治政府建设取得实效。公共资源交易中心建设启动，信息公开和办事公开范畴扩大。强化行政执法监察，软环境明显优化。加强绩效评估和调度督导，行政执行力和工作效能提升。创新社会管理，195个社区全部推行网格化管理；严格落实各项制度和措施，全社会的安全稳定局面得到了有力维护。

　　各位代表，五年来取得的辉煌成就，是市委总揽全局、团结带领全市干部群众，用辛劳和智慧、激情与拼搏，谱写的全市发展史上的精彩华章。今天的北国江城，正在豪情满怀地迈向东北和东北亚重要的新型产业基地、旅游度假名城和生态宜居城市！在此，我代表市政府，向全市450万人民，向辛勤工作在各条战线的广大建设者，向各级人大代表、政协委员、各民主党派、工商联和社会各界人士，向中省直单位、驻吉部队指战员和武警官兵，向始终关心支持吉林市发展的海内外朋友，表示衷心的感谢和崇高的敬意！

　　回顾五年的奋斗历程，我们更加深刻地认识到，推动吉林市现代化建设事业不断前进，必须在党的领导下，始终坚持科学发展，在加快发展中转变方式，在扩大总量中优化结构，努力走出具有我市特色的又好又快发展新路子；始终坚持以人为本，做到经济增长与民生改善共进，发展成果由广大人民共享，真正让城乡居民生活得更加美好，为改革发展凝聚起更广泛的群众基础；始终坚持思想解放，用改革的办法、创新的精神、开放的姿态，着力完善有利于科学发展的体制机制，破解掣肘科学发展的矛盾问题，为经济社会发展注入强大的生机和动力。

　　同时，我们也清醒地看到，发展中还存在一些差距和不足。一是综合实力与富民强市的要求不相适应。总量不足、结构不优、开放度不够的问题还没有从根本上解决，欠发达仍是我市现阶段的基本市情。二是民生改善和提高步伐与人民群众的热切期盼不相适应。城乡居民收入整体水平还不高，仍有部分群众生活困难，改善民生还需进一步加大力度。三是一些领域行政效能与新形势新任务的需要不相适应。个别部门和干部解放思想程度不够、创新意识和服务理念不强、效率不高等问题仍然不同程度地存在。对于这些问题，我们将以高度负责的精神，认真加以解决。

　　二、今后五年重点发展任务

　　未来一个时期，是我市全面建成小康社会的决定性阶段。我们一定深入贯彻党的十八大、省十次党代会和市十二次党代会精神，以“三年看吉林、实现新跨越”为战略基点，全力推进今后五年经济社会发展。总的要求是，始终坚持科学发展的主题，把握加快转变发展方式，统筹推进新型工业化、信息化、城镇化和农业现代化的主线，牢牢抓住发展和民生两个关键，突出十大功能区和县域发展两个重点，力争在“十二五”末提前完成省、市党代会提出的两翻番、双倍增、七跃升和五个新跨越目标的基础上，继续推进又好又快发展，全力建设东北和东北亚重要的新型产业基地、旅游度假名城和生态宜居城市，为在全省率先实现全面建成小康社会目标打下坚实基础。

　　到2017年，地区生产总值迈上6000亿元新台阶，地方级财政收入达到300亿元，综合竞争力在全国地级以上城市排位前移5位以上；三次产业结构调整为5：51：44，初步实现老工业基地向新型产业基地的转换；中心城市建成区扩大到210平方公里以上，现代化大城市框架基本构成；单位GDP综合能耗下降15.2%，主要污染物排放总量显著减少，生态城市建设水平全面提升；城镇居民人均可支配收入、农民人均纯收入年均增长15%以上，处于全省领先水平。

　　——建设现代产业体系，全力打造新型产业基地

　　按照“稳定一产、优化二产、提升三产”的思路，推进产业结构战略性调整，全面增强经济发展的协调性和竞争力。

　　加快传统产业改造升级。采用先进技术改造支柱优势产业，着力延伸产业链条，提高集群化、精细化、终端化水平。石化产业，大力发展精细化工，建成国际一流的综合性化工基地，到2017年，销售收入突破2800亿元。汽车产业，具备百万辆整车生产能力，跃上千亿级产业规模新台阶。冶金产业，围绕黑色、有色、炉料三大主线，做强精品钢，深度开发镍、钼等优势矿种，销售收入向千亿元迈进。农产品加工产业，以中新吉林食品区建设为示范，快速打造具有国际竞争力的安全优质食品基地。坚持特色化、品牌化方向，加大能源、轻纺、非金属矿产开发转化力度，提高资源产出效益，再造产业发展新优势。

　　大力培育战略性新兴产业。突出具有比较优势和成长潜力的重点领域，着力发展以碳纤维及制品为核心的新材料产业，以核电风电装备、大型专用设备为标志的先进装备制造业，以电子元器件、工控电子为代表的电力电子产业，以生物医药、生物化工为特色的生物技术产业，以油页岩综合利用、生物质能为重点的新能源产业，推进战略性新兴产业系统化、规模化发展，积极抢占未来产业发展制高点。到2017年，战略性新兴产业占规模工业产值比重达到32%。

　　促进现代服务业快速发展。统筹发展市区的河南街、东市和江南等核心商圈，着力建设滨江商业带、特色商业街、城市综合体、大型批发市场群，完备提升区域性商业中心功能，全面增强商贸流通业辐射力和服务品质。坚持走文化旅游融合发展之路，整体布局，市县联动，深度挖掘历史、山水、人文和特色文化资源，综合开发松花湖、白山湖、龙潭山、莲花山等重点景区，加快建设高档酒店等旅游配套设施，努力打造独具魅力的我国北方休闲度假旅游养生目的地。积极发展第三方物流，科学规划布局化工、汽车、冶金等专业物流基地，推进一批大型综合物流园区和县（市）级物流节点建设。大力发展信息服务业和服务外包产业，建设国内重要的“云计算”平台和省内最大的服务外包基地。推动金融改革创新，引进发展各类金融机构、金融业态和金融衍生品，拓展融资渠道，扩大金融总量，争取5户以上企业首发上市。鼓励支持法律、会计、资产评估等市场中介组织发展。

　　推进农业现代化进程。以建设现代农业产业体系为重点，深化农业结构调整，形成优势突出、特色鲜明的农产品产业带。发展土地集约经营，加强农业基础设施建设，粮食产量力争突破120亿斤。建成标准化养殖小区1600个，畜禽规模化养殖率提高到86%。发挥资源优势，开发一批在国内外市场具有较大影响力的长白山特色品牌产品。强化龙头企业带动，提升产业化经营水平，主要农产品加工转化率达到85%以上。

　　——推进特色城镇化，全力构筑现代化大城市框架

　　把城镇化作为战略突破口，加大统筹城乡发展力度，有效形成城乡共同繁荣的区域发展新格局。城镇化率提高到64%。

　　推动中心城市提质扩容。优化“北工、中商、南居”空间结构形态，具备250万以上人口的承载能力。十大功能区年均完成基础设施投入100亿元、产业项目投入600亿元，2017年实现生产总值占市区50%以上，高新南区、经济开发区、化工循环经济示范园区、金珠工业区、北大壶开发区进入成熟发展阶段，高新北区、南部新城、松花湖旅游区、中新吉林食品区空间框架基本形成，哈达湾区域工业企业搬迁全部完成。加大旧城改造力度，成片棚户区、危旧房、“城中村”改造基本完成。完善综合交通运输体系和公共交通服务体系，全线贯通“八横八纵”主干路网，建设10座跨江大桥，有效解决城市交通拥堵问题；拓展对外通道，建成吉珲客运专线、铁路西环线、吉哈高速、长吉高速拓宽及南北线改造，二台子机场实现复航。加强生态文明建设，以节能减排财政政策综合示范城市建设为契机，推行绿色、循环、低碳生产生活模式，集约节约利用资源，加大生态系统建设和环境保护力度，着力打造天蓝、地绿、水净的美丽家园。

　　加快县级城市和小城镇建设。强化产业支撑，完善城市功能，全面提升县级城市的发展能力和形象品位，磐石、桦甸、蛟河、舒兰建成区面积均在25平方公里以上，人口达到或接近中等城市规模，永吉与市区对接发展、逐步融合，5县（市）全部进入全省县域发展综合实力15强。坚持统筹规划、梯次推进，促进一批重点城镇向小城市发展，加快建设一批中心镇、特色镇，带动以农民新型居住社区建设为重点的新农村发展，基本形成布局合理、特色突出、功能互补的小城镇体系，城镇基础设施配套完善，公共服务体系基本健全。

　　——切实保障和改善民生，全力建设幸福吉林

　　人民群众的需要就是我们的工作目标。更加注重社会公平正义和百姓内心的幸福感受，让城乡居民学有所教、劳有所得、病有所医、老有所养、住有所居，真正做到体现民意、惠及民利、赢得民心。

　　提高城乡居民收入水平。实施就业优先战略和更加积极的就业政策，完善公共就业服务体系，促进创业带动就业，实现城乡更高质量的就业。合理调整分配关系，突出抓好农民、企业职工、中低收入者和困难家庭增收，促进城乡居民工资性收入稳步增长，多渠道增加经营性、转移性和财产性收入，努力实现居民收入增长和经济发展同步、劳动报酬增长和劳动生产率提高同步。

　　健全社会保障体系。推进各项社会保险由制度全覆盖向人群全覆盖延伸，逐步提升统筹层次和保障水平，进一步增强社会保险基金抗风险能力，减轻参保人员负担。完善城乡低保标准自然增长机制，积极发展社会救助、社会优抚、社会福利和慈善事业。加强防灾救灾体系建设。培育壮大养老产业，提高养老服务社会化水平。加大保障性住房和暖房子工程建设，城镇和农村人均住房面积分别达到32和31平方米；建成我国北方第一个全城暖房子的城市。

　　推进基本公共服务均等化。坚持教育优先发展，统筹各级各类教育协调发展，扩大优质教育资源，提高教育质量，促进教育公平，努力办好人民满意教育。深化医药卫生体制改革，健全覆盖城乡的公共卫生服务体系，实现人人享有基本医疗卫生服务。加强公共文化设施建设，增强文化产品生产供给能力，扩大中国历史文化名城对外影响力。发展体育事业，落实全民健身计划纲要，提高全民身体素质。支持国防和军队建设，拓展军民融合发展内涵，实现全国双拥模范城八连冠。

　　——强化要素保障，全力构建科学发展支撑体系

　　以开放为先导，以创新为动力，以人才为引领，以环境为保障，全面营造开放包容、创新突破、人才兴业、环境立市的发展氛围，为科学发展、加快振兴提供强有力的支撑。

　　深入扩大对外开放。实施更加积极主动的开放战略，更宽领域、更深层次、更高水平参与国内外分工，海内外产业转移进驻速度、资本投入强度明显提升，5年引进跨国公司90户，累计招商引资到位资金6430亿元。准确把握政策取向，强化“上争”机制，最大程度获得国家和省的支持。优化出口结构，培育外贸经营主体，多元开拓国际市场。推进区域合作，扩大与长吉图开发开放先导区、图们江区域、罗先经贸区和东北其他城市的经济合作范畴，长吉一体化取得重大进展。

　　加大改革创新力度。以市场化改革为取向，着力在行政管理、财税金融、社会事业及功能区管理体制等重点领域和关键环节取得重大突破，系统完备、科学规范、运行有效的制度体系有效构建。强化企业在技术创新中的主体地位，健全产学研政合作机制，突破一批核心和关键技术，打造一批拥有自主品牌、较强竞争力的高新技术企业集群，科技创新对经济增长的贡献率大幅提升。

　　加快构建人才高地。牢固树立人才是第一资源的理念，搭建人才创业平台，完善政策服务体系和选人用人机制，以高层次人才和紧缺人才为重点，着力引进培养一批急需的企业经营管理人才、科技领军型人才和一线实用专业技术人才，优化人才结构，激发人才潜能，努力建设一支规模较大、结构合理、素质优良的人才队伍，使我市成为创新人才的聚集地。

　　大力改善发展环境。在加大硬环境建设的同时，把软环境建设摆在更加突出的位置，深入转变政府职能，深化审批制度改革，规范行政执法行为，提高政务服务质量，严厉惩处破坏软环境的行为，努力建设办事环节最少、办事效率最高、办事成本最低、投资者和群众最满意的投资创业环境，吸引更多的资源要素进入并留驻我市。

　　三、明年工作安排

　　2013年是新一届政府的开局之年，也是“三年看吉林、实现新跨越”的起步之年。我们要以实施十项攻坚战为核心，加大突破力度，全面推进经济社会又好又快发展。

　　主要预期目标：地区生产总值同比增长19%，规模工业增加值增长20%，招商引资到位资金增长23%，全社会固定资产投资增长28%，社会消费品零售总额增长20%，地方级财政收入增长22%，城镇居民人均可支配收入、农民人均纯收入分别增长16%和18%。

　　（一）加快功能区建设和县域经济发展，切实打牢科学发展的载体支撑

　　强化政策支持，创新体制机制，落实各项发展条件，推进十大功能区实现新的跨越。实施基础设施项目100项、重点产业项目350项，完成地区生产总值、地方级财政收入占市区总量的42%。高新南区，突出汽车及零部件、装备制造、信息服务产业发展，组织实施一汽吉林扩能改造G4、T系列发动机、8英寸芯片等一批重点项目。高新北区，创新建设运作模式，拓展融资渠道，完成5平方公里征地拆迁，加快吉星汽车轮胎、东忠服务外包等项目实施进程。经济开发区，启动西区和北区6平方公里开发，着力建设碳纤维特色产业园、聚酰亚胺树酯等重点项目，快速形成碳纤维、精细化工产业集群发展新态势。化工循环经济示范园区，深化国际化合作，以延伸丙烯产业链为重点，加大30万吨环氧丙烷、25万吨双氧水、12.8万吨石油树脂等项目建设步伐。金珠工业区，积极承接中心城区产业转移，推进钢铁产业园、物流园区建设，开工100万吨冷轧钢、30万吨焊管等重点项目。哈达湾服务业集中区，完成晨鸣纸业、冀东水泥搬迁，推进中钢吉铁、中钢吉炭企业搬迁前期工作，实施棚户区改造和高标准回迁安置小区建设。南部新城，完善相关规划，加快欧亚城市综合体、卫校等服务业和社会事业重点工程建设，恢复小白山望祭殿，建设满族文化公园，着力打造滨水宜居生态新城。松花湖旅游区，推进万科松花湖国际旅游度假区2平方公里旅游小镇、朱雀山综合开发等项目建设，松花湖滑雪场改造投入运营，延伸联动开发沿江水上旅游线路。北大壶开发区，围绕打造中国北方国际休闲旅居度假区，加快建设桥山、保利、旭飞等旅游综合开发项目。中新吉林食品区，完成与淡马锡合资平台公司组建，建设国际物流园、中新成生猪产业化、中信有机水稻系列精深加工等一批重点项目，加快向国家级开发区迈进。

　　围绕资源型城市转型，突出县域开发区建设，强化产业支撑，推进县域经济结构战略性调整，县域生产总值、地方级财政收入分别增长20%和22%。永吉，以“两区”建设为核心，增强大黑山钼业、冀东水泥等骨干企业带动功能，引进建设上下游配套产业项目，切实提高产业集中度和投资强度。舒兰，快速提升“一区三园”承载能力，抓好小城钼矿开发、大润德农产品综合开发等一批重点项目，全面加快经济转型步伐。磐石，加大一汽铸造基地、亚融电池基地等项目建设支撑力度，壮大主导产业优势，促进现代医药、新材料等新兴产业不断成长，快速向东北新型工业城市迈进。蛟河，围绕实现三个“30”目标，统筹推进蛟河新区、天岗石材产业园等领先发展，加快蛟河国际内陆港、500万吨洗煤深加工等重点项目建设速度。桦甸，全力打造新能源、新材料、金属矿产及加工三个百亿级产业，推进桦甸经济开发区及产业园区建设，有序推动资源整合和集约开发，力争早日进入东北三十强县行列。

　　（二）强化项目建设和招商引资，切实增强可持续发展后劲

　　全力抓好固定资产投资和项目建设。实施3000万元以上重点项目1200项，其中亿元以上项目650项；竣工3000万元以上重点项目400项。突出项目谋划，包装、储备一批规模大、技术先进、效益明显的重点项目，储备亿元以上项目200项，项目实施率达到30%以上。突出产业结构优化升级，建设3000万元以上工业项目600项，建成投产200项；服务业项目投资比重提高到34%。突出项目管理，抓好160项5亿元以上重点项目，着力推进10亿元以上重大产业工程；健全项目调度督导和考核激励机制，强化项目前期手续办理、集中开工、巡检讲评，有力保障项目建设的顺利进行。

　　着力做好招商引资工作。一是扩大招商成果。坚持政府推动和企业主体招商相结合，突出专业招商、节会招商、以商招商等方式，下力气引进一批重大项目，开工3000万元以上招商项目350项，其中亿元以上项目160项。二是提升招商质量。注重产业链延伸、科技含量、产出效益和生态影响，集中攻关世界500强、跨国公司、大型央企和行业领军企业，促进引资结构的进一步优化。三是提高招商服务水平。健全各县（市）区、功能区之间协调合作机制，合理统筹项目布局，推进利益共享，努力实现招商效益的最大化；畅通绿色通道，实施招商项目全程跟踪服务，切实提高项目履约率、开工率和资金到位率。

　　（三）着力推进新型工业化，切实壮大产业发展实力

　　努力提高工业经济发展水平。大力推进工业提速增效，规模工业总产值、利润均增长20%。一是实施传统产业改造升级和战略性新兴产业跃升计划。提高技改投资比重，发展精深加工和协作配套，支柱产业占规模工业产值达到68%；突出抓好85项3000万元以上战略性新兴产业项目，战略性新兴产业产值增长25%。二是促进产业配套融合。围绕乙烯、丙烯产业链开发，积极发展聚烯烃、合成树脂、合成橡胶等上下游产业；立足汽车发动机、汽车专用料、车用化学品等产业链衔接，组织实施1500万条轮胎、10万吨改性汽车专用料等项目；引进关键技术，培育发展为骨干企业、重大项目配套的加工企业，提高原料本地配套深加工比例。三是推进现有企业做大做强。加大工业运行监测分析，加强要素保障服务，实施阶梯式扶持政策，推动企业扭亏增盈，促进以百户企业为重点的规模工业企业快速发展。百户企业产值增长18%以上。四是全力实施民营经济壮大工程。出台促进民营经济发展实施意见，加强管理咨询、创业辅导等公共服务平台建设，为民营经济发展营造良好的服务环境；实施民营企业梯次培育计划，推动民营经济总量上规模、质量上水平，新增规模工业企业100户，产值过亿企业发展到500户；深入开展全民创业，激发民间资本创业热情，年内新增小微企业1000户，个体工商户10000户。五是强化自主创新。加强品牌培育和质量管理，开展第二届市长质量奖评选活动，着力打造一批品牌名牌产品；推进100种重点新产品规模化生产，快速形成市场竞争新优势。六是有效盘活存量资产。采取重组合作、技改投入、出租出售等方式，提高停产企业开工率、半停产企业装置负荷率，盘活存量资产和闲置土地，努力实现资产效益最大化。七是抓好建成项目增产达效。分类施策，协调落实生产条件，促进去年以来建成投产项目有效发挥产出效能，达产达效率力争达到80%。八是加强企业家队伍建设。发挥企业家协会、联合会作用，搭建交流合作平台，强化中高级企业管理人员培训，努力造就一支懂经营、会管理、善决策、能创新的企业家队伍。

　　大力推进服务业跃升发展。服务业增加值增长20%以上。一是提升商贸流通业。抓好东方伟业综合体、悦榕庄酒店等68个商贸重点项目建设；改造升级东北亚农产品批发市场等现有市场，加快亚琦综合市场、众安居家居广场等大型市场建设步伐，切实提高商贸经济辐射集散能力。扶持商贸企业发展壮大，培育销售额10亿元以上企业5户、5亿元以上10户、亿元以上25户，限上商贸企业突破1300户。二是努力发展文化旅游业。充分挖掘以一座龙潭山、半部东北史为主题的历史文化，以一江、一湖、四山为主题的山水文化，以陨石、雾凇、滑雪、温泉、避暑为主题的特色文化，以北山、佛手山为主题的人文文化，设计包装区域文化旅游精品线路，深度开发文化旅游产品，推进节庆、赛会、旅游交互融合，旅游收入增长25%。三是着力发展生产性服务业。加快增益物流园、香港健坤商贸物流园、吉高陆港物流园等项目建设步伐，荣德钢铁、万森化工燃气等物流项目尽快投入使用。着力建设中油数据中心、软银数据中心等重点项目，推进信息服务业和服务外包产业快速发展。生产性服务业占第三产业增加值比重达到53%。

　　（四）强力推动特色城镇化，切实构建城乡协调发展新格局

　　大力抓好以十大民心工程为核心的城市基础设施建设。完成城建投资258亿元。全面推进西部快速路、东部快速路、雾凇西路、高新大路、松花江堤防、吉林大桥畅通、迎宾大路立交桥、西关立交桥、吉林站西广场、朱雀山公园开发10大民心工程建设。新建红旗大桥、新城大桥。组织实施长吉南线改造、吉哈高速、铁路西环线等一批重大交通项目。启动六水厂建设，完成污水处理厂二期和三水厂二期工程。建设改造供水管线1700米、天然气管网25公里、供热管网100公里。加大旧城区改造步伐，推进房地产业健康发展，开发850万平方米，竣工500万平方米。完善新建小区配套设施建设。

　　实施数字城市建设。搭建数字化政务平台，扩大网上办公领域，提高政务服务效率。搭建数字化商务平台，实施物流企业电子商务工程。搭建数字化服务平台，加快教育、医疗、防汛抗旱等社会领域信息系统建设。搭建数字化管理平台，城市规划实现“一张图”管理，建设城市地理信息系统、智能交通管理系统和社区管理服务应用系统。

　　强化城市管理。加强规划编制，严格规划立法和执行程序，强化规划监管。实施市容管理标准化建设，组织开展牌匾广告、散流体等专项整治行动；移植乔木5万棵，栽植花卉260万株；对主街路临街建筑物“穿衣戴帽”；启动雾凇大桥、江城大桥亮化工程，完成北山景区主题亮化改造。健全小区物业长效管理机制，全面推行物业标准化管理。完善垃圾清运体系，推进垃圾分类收集和垃圾处理产业化。优化道路交通秩序，持续开展乱停乱放专项整治，加快建设各类停车场，切实保障交通畅通。开展文明城市建设，提高市民文明素质，营造全市共同参与城市管理的社会氛围。

　　推进县级城市和小城镇建设。加强水、电、气、热、道路等基础设施建设和管护，完备教育、文化、卫生等社会事业功能，提高公共服务能力和人口吸纳水平，新增建成区7平方公里、城镇人口5-7万人。将孤店子、红石等18个重点镇作为城镇化试点，实施简政放权，鼓励先行先试，在土地流转、促进农民进城等方面取得实质性突破；试点镇经济总量、财政收入分别增长20%和25%。启动第二轮千村示范、万村提升工程，建设示范村140个。

　　（五）积极发展农业现代化，切实建设现代农业产业体系

　　一是推进粮食生产集约化。搞好高标准基本农田建设，稳定粮食播种面积，粮食产量保持110亿斤阶段性水平。二是积极发展畜牧业和园艺特产业。新建扩建200个标准化规模养殖小区，畜牧业产值增长9%。推进桦甸二道甸子国家级野山参等5个标准化示范园区建设；深入实施“菜篮子”工程，再建温室大棚2000栋，园艺特产业产值增长22%。加大林业生态整治力度，退耕还林1.5万公顷。三是加快农业产业化进程。建设永吉绿色稻谷精深加工、蛟河速冻玉米及食用菌加工等一批农业产业化重点项目，整合打造精品大米等农产品品牌，市级以上龙头企业发展到220户，农产品加工销售收入增长18%以上。四是夯实农业发展基础。组织建设水利基础设施418项，农业综合机械化水平达到69%，新增村级信息服务站100个，农业保险参保面积占农村土地承包面积的85%，农业科技贡献率达到57%。继续实施农村安全饮水工程，再解决13.7万人饮水安全问题。五是深化农村综合改革。稳步推进土地流转，土地流转面积占承包土地面积比重提高到12%；完善失地农民就业、保障制度；抓好乡村公益性债务化解工作；新发展农民专业合作社300个。

　　（六）努力抓好财税金融工作，切实提升对经济社会发展的支撑能力

　　加强财税征管组织，健全财税增收机制，充分挖掘增收潜力，形成组织收入的工作合力；加大属地纳税征管力度，着力解决一些企业纳税地转移等问题。深化社会综合治税，完善综合治税信息共享平台，建立健全涉税信息指标体系、信息采集机制和监督考核机制，进一步提高对税源的掌控能力；深入推进个体税收社会化管理，扩大街道、社区零散税收委托代征范围。加强国有企业经营目标考核和绩效管理，完善国有资产监督管理体制，确保国有资产资源保值增值。发挥各县（市）区、开发区发展总部经济的主体作用，引进年纳税超1000万元总部经济企业10户以上。推行预算绩效管理试点，实施公务卡管理制度；强化预算编制、预算执行和决算监督管理。加强地方政府性债务管理，防范和化解债务风险；做好“营改增”试点准备工作。

　　修订完善银行业金融机构考核奖励办法，支持银行业优化信贷结构，增大对实体经济和中小微企业的支持力度，有效扩大信贷规模，存贷比力争达到全国平均水平。引进发展股份制银行、村镇银行和专业创业投资机构，新设小额贷款公司、担保公司等非银行业金融机构15户以上，积极培育各类金融中介组织。提升政府性融资平台治理水平和盈利能力，支持市中小企业担保公司增资扩股，健全都邦保险内部治理结构。积极发展资本市场，推动城投、铁投等企业发行债券，大力发展股权投资基金，加快昊宇电气、科龙建筑、西点药业、康乃尔化工上市步伐，组织实施国企上市的创新实践。

　　（七）认真办好民生事项，切实扩大改善民生工作成果

　　将年度新增财力的80%用于保障和改善民生，组织实施十大方面100项民生事项，重点有：

　　1．千方百计扩大就业。城镇新增就业9万人以上，应届普通高校毕业生年末统计就业率达到90%，农村富余劳动力转移就业65万人次，城镇登记失业率控制在4%以内，“零就业”家庭保持动态为零。

　　2．加强医疗保障体系建设。加快人民医院二期、中心医院门诊综合楼等一批重点项目建设；5个县级医院全部新建并达到二甲水平，60%村卫生室、70%乡镇卫生院达到标准化要求；城镇居民医疗保险、新农合报销最高支付限额分别提高到6.5万元和9万元；参保城镇居民在一、二、三级医院住院报销比例分别提高到85%、70%和55%；新农合参保率达到99%以上，乡镇卫生院和县级医院报销比例分别提高到85%和75%；符合救助条件的困难群众住院治疗自理费用平均降低到20%以内。

　　3．继续实施安居工程。建设廉租住房1500套、7.3万平方米，公租房830套、5万平方米；完成棚户区（危旧房）改造征收50万平方米，开工240万平方米；加快解决低保无房、低收入无房家庭住房困难；完成1000万平方米暖房子工程改造任务。

　　4．进一步提高社会救助标准。城乡居民最低生活保障标准分别达到上年度城镇居民人均可支配收入的20%和农民人均纯收入的25%以上，保障标准和补差水平位于全省前列；再解决1万户城市低保家庭再保障问题。

　　5．扎实推进低碳城市建设。围绕产业低碳化等“六化”建设，实施节能减排示范项目221项；城区集中供热普及率提高到94%；加强空气污染治理，空气质量好于二级以上天数保持在85%以上。

　　6．加强水源地保护及污水治理。扎实推进松花江污染综合防治，启动城区雨排水管网改造，加大工业点源和农业面源污染治理力度，城市饮用水水源地水质达标率保持100%，松花江白旗出境断面水质稳定达到三类标准。

　　7．实施街路畅通便民工程。对北京路、朝阳街、青岛街等14条主要街路进行拓宽改造，提高现有道路通行能力。新建4处公交运营车场及一批港湾式停车站和回转车场，再更新公交车辆300台；扩大公交线网覆盖密度，增加发车班次，延长营运时间，方便市民出行。

　　8．构建完善养老服务体系。积极发展养老产业，实现县（市）区级社会福利服务中心、社区居家养老服务站全覆盖，新增机构养老床位3000张以上；加强农村社会福利服务中心建设，将符合条件的农村五保对象全部纳入保障范围。

　　9．治理教育“三乱”问题。深入开展中小学校和教师乱办班、乱补课、乱收费集中整治活动，规范幼儿园收费，治理义务教育阶段择校收费，严格执行公办普通高中择校生“三限”政策，加强民办教育违规办学整治。

　　10．提高社会公众安全感。强化生产、消防、交通、燃气、食品和建筑等领域安全管理，推进肉类蔬菜流通追溯体系建设，严控重大安全生产事故发生；严厉打击各类犯罪行为，全市刑事案件实现稳中有降。

　　（八）大力繁荣社会事业，切实推进经济与社会协调发展

　　推动文化大发展大繁荣。加快建设全民健身中心、人民剧院等大型文体项目，完善乡镇文化站、社区文化活动中心等基层文化设施功能，深入实施广播电视村村通工程，启动城市社区书屋建设。办好第三届松花江河灯文化节，广泛开展多种形式的群众文体活动，进一步丰富城乡居民文化生活。深化文化体制改革，推进影视制作、演艺娱乐、出版发行、艺术品交易等优势文化产业和动漫、创意等新兴文化产业快速发展，扶持演艺集团、松花江文化发展公司等市场主体做大做强，组建文化产业投资集团，提高文化产业规模化、专业化、特色化发展水平。

　　加大科技创新步伐。加快科技创新平台建设，新增市级企业技术中心20家，培育市级技术创新团队20个。筹建先进装备制造、生物医药技术创新等战略联盟。加强产学研合作，深入开展“校地合作”。实施“百名专家服务百户企业，解决百项技术难题”工程，鼓励科研人员服务技术改造和产业升级。加快科研成果转化及产业化步伐，实现科技成果转化50项。

　　推进教育、卫生事业发展。每个乡镇至少办好一所公立中心幼儿园，有效缓解“入园难”问题；加大薄弱校改造步伐，推进基础教育均衡发展；创建3个职教集团，启动职教园区建设；支持驻吉高校发展。深化医药卫生体制改革，推行医保付费谈判机制，全面推进县级公立医院改革；抓好农村三级网络标准化建设，提高城市社区卫生服务中心和区域医疗中心服务水平；加大医疗药品市场监管，规范医疗服务行为。做好人口和计生工作。

　　（九）创新社会管理，切实加大和谐社会建设步伐

　　完善社区网格化管理服务机制，开展社区网站试点建设，畅通社情民意绿色通道，积极推进标准化社区创建活动；提升社区工作人员整体素质，支持社区志愿者工作，健全社区服务体系，逐步满足社区居民的多样化、个性化生活需求。加强社会组织监督管理，引导社会组织健康有序发展。开展“信访积案化解年”活动，严格落实领导接访下访、领导包案和首办负责制，完善人民调解、行政调解、司法调解协调联动的工作体系；以征地拆迁、涉法涉诉、社会保障等领域为重点，妥善处理人民群众合理诉求，推进矛盾纠纷排查调处的经常化、制度化和即时化。完善社会治安防控体系，加强公共安全体系和企业安全生产基础建设，加大应急管理科技支撑力度，切实保障人民群众生命财产安全。做好民族宗教工作，保持民族宗教领域和谐稳定。深入开展双拥共建活动，认真落实优抚安置新政策，努力解决驻吉部队军官家属实际困难，巩固和发展军政军民团结。

　　（十）加强政府自身建设，切实打造人民满意政府

　　坚持科学理政。认真落实重大事项向市委请示、向人大报告和政协通报制度，自觉接受人大的法律监督、工作监督和政协的民主监督。把调查研究作为谋事之基、成事之道，坚持深入基层问政于民、问需于民、问计于民。完善决策程序，健全重大问题公众参与、专家论证、风险评估、合法性审查和集体决策机制，不断提升科学决策、民主决策、依法决策水平。狠抓决策落实，提高政府执行力，坚决纠正和查处有令不行、有禁不止的现象和问题。

　　推进依法行政。贯彻落实全市依法行政规划，把依法行政贯穿于政府工作的全过程。政府工作人员带头学法、讲法、守法，增强法律意识，倡导尊重法律的社会风尚。完善立法工作程序，提高政府立法质量，使制度建设更加符合经济社会发展的客观需要。严格法定权限和程序，规范行政执法自由裁量权，强化监督和问责，促进执法行为公正、文明、规范。开展法治江城建设，深入做好六五普法工作，提高全社会法治化水平。

　　优化政务环境。大力推进行政管理体制改革创新，坚决做到“五个一律”：进一步推进政事分开，凡是可以取消的收费一律取消，把政府部门所属的评估、公证、交易等中介机构分离出去；深化行政审批制度改革，凡是可以精简的审批一律精简，即办事项达到50%以上，承诺事项提前办结率突破90%；合理划分事权，凡是可以下放的权力一律下放，解决好市与县（市）区之间职权不合理、责权不统一问题；完善政务公开，凡是能公开的事项一律公开，让权力在阳光下运行；规范公共资源交易行为，凡是涉及建设工程招投标、土地使用权出让、政府采购、国有产权交易等一律进入公共资源交易中心操作。

　　加强从严治政。严格执行中央关于改进工作作风、密切联系群众的八项规定，大力开展下基层、转作风、促发展、惠民生活动。改进会风文风，开短会、讲短话，严控会议数量规模，注重会议实效；规范精简各类文件，清理规范简报、信息。科学安排公务活动，轻车简从、减少陪同、简化接待。未经批准，各县（市）区、各部门不得举办庆典、研讨会、论坛等活动。从各级政府班子做起，进一步强化大局意识、协作精神、务实作风，力戒空谈浮躁，力戒慵懒散软，着力在“快、实、细、新”上下功夫，加快工作节奏，做实工作措施，细化工作组织，创新工作路数，把更多精力投放到为发展攻坚破难、为人民做事谋利上来，实实在在地提高发展质量。认真落实党风廉政建设责任制，加大源头治腐力度，严厉查处违法违纪案件和破坏软环境行为，坚决纠正损害群众利益的不正之风，全面塑造清正廉洁、干净干事的政府形象。

　　各位代表，未来发展的蓝图已经绘就。我们一定在市委的正确领导和市人大、市政协的大力支持下，进一步解放思想，改革开放，凝聚力量，攻坚克难，全力建设开放吉林、法治吉林、文明吉林、幸福吉林，在全省实现率先发展，向着全面建成小康社会的宏伟目标加速迈进！
